# Constructing Chromium Multioxide Hole‐Selective Heterojunction for High‐Performance Perovskite Solar Cells

**DOI:** 10.1002/advs.202203681

**Published:** 2022-08-28

**Authors:** Sheng Jiang, Shaobing Xiong, Wei Dong, Danqin Li, Yuting Yan, Menghui Jia, Yannan Dai, Qingbiao Zhao, Kai Jiang, Xianjie Liu, Liming Ding, Mats Fahlman, Zhenrong Sun, Qinye Bao

**Affiliations:** ^1^ School of Physics and Electronic Science East China Normal University Shanghai 200241 China; ^2^ Shanghai Key Laboratory of Magnetic Resonance East China Normal University Shanghai 200241 China; ^3^ State Key Laboratory of Precision Spectroscopy East China Normal University Shanghai 200241 China; ^4^ Laboratory of Organic Electronics, ITN Linköping University Norrköping SE‐60174 Sweden; ^5^ Center for Excellence in Nanoscience (CAS), Key Laboratory of Nanosystem and Hierarchical Fabrication (CAS) National Center for Nanoscience and Technology Beijing 100190 China; ^6^ Collaborative Innovation Center of Extreme Optics Shanxi University Taiyuan Shanxi 030006 China

**Keywords:** charge transport, efficiency, hole‐selective heterojunction, nonradiative recombination, perovskite solar cells

## Abstract

Perovskite solar cells (PSCs) suffer from significant nonradiative recombination at perovskite/charge transport layer heterojunction, seriously limiting their power conversion efficiencies. Herein, solution‐processed chromium multioxide (CrO_x_) is judiciously selected to construct a MAPbI_3_/CrO_x_/Spiro‐OMeTAD hole‐selective heterojunction. It is demonstrated that the inserted CrO_x_ not only effectively reduces defect sites via redox shuttle at perovskite contact, but also decreases valence band maximum (VBM)‐HOMO offset between perovskite and Spiro‐OMeTAD. This will diminish thermionic losses for collecting holes and thus promote charge transport across the heterojunction, suppressing both defect‐assisted recombination and interface carrier recombination. As a result, a remarkable improvement of 21.21% efficiency with excellent device stability is achieved compared to 18.46% of the control device, which is among the highest efficiencies for polycrystalline MAPbI_3_ based n–i–p planar PSCs reported to date. These findings of this work provide new insights into novel charge‐selective heterojunctions for further enhancing efficiency and stability of PSCs.

## Graphical abstract

Solution‐processed chromium multioxide hole‐selective heterojunction boosts power conversion efficiency and stability of perovskite solar cells.

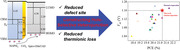

## 1 Introduction

Organic–inorganic halide perovskite semiconductors have received much attention as one of the most auspicious next‐generation photovoltaic absorbers due to their superior optoelectronic properties including large absorption coefficients,^[^ ^1^, ^2^, ^3^ ^]^ small exciton binding energies,^[^ ^4^, ^5^ ^]^ and high carrier mobilities.^[^ ^6^, ^7^, ^8^ ^]^ Attribued to worldwide research efforts recently, the past decade has featured a fantastic growth rate in the power conversion efficiency (PCE) of single‐junction perovskite solar cells (PSCs) from 3.8%^[^ ^9^ ^]^ to a certified 25.7%,^[^ ^10^ ^]^ which approaches the efficiencies of state‐of‐the‐art crystalline silicon solar cells. However, the “imperfect contact” between the perovskite and charge transport layers within PSCs seriously limits further improvement in both efficiency output and operational stability.^[^ ^11^, ^12^ ^]^ It is well established that polycrystalline perovskite films inevitably suffer from tremendous defects on the film surface,^[^ ^13^ ^]^ which cause nonradiative recombination of photogenerated carriers and Fermi level pinning at interface.^[^ ^14^, ^15^, ^16^ ^]^ Moreover, the interface energetics at the contact have a very important influence on the device performance, especially in terms of photovoltage.^[^ ^17^ ^]^ The relative positions of perovskite's valence or conduction bands and charge transport layer's frontier energy levels, i.e., highest occupied molecular orbital (HOMO) or lowest unoccupied molecular orbital (LUMO) levels, significantly determine charge transport across the interface.^[^ ^18^, ^19^ ^]^ Therefore, constructing optimized charge‐selective heterojunction with reduced defects and matched energetics will be crucial to further improve the performance of PSCs.

The solid‐state 2, 2', 7, 7″‐Tetrakis[*N*,*N*‐di(4‐methoxyphenyl)amino]‐9, 9″‐spirobifluorene (Spiro‐OMeTAD) is the most widely used hole transport layer in high‐performance PSCs with n–i–p configuration.^[^ ^20^, ^21^ ^]^ However, the existence of a large energy level offset between perovskite's valence band maximum (VBM) and Spiro‐OMeTAD's HOMO constrains the built‐in field and leads to theromionic loss for the collected holes at the heterojunction in the PSCs.^[^ ^22^, ^23^, ^24^, ^25^, ^26^ ^]^ Furthermore, the penetrative iodine ions (I^–^) can chemically interact with Spiro‐OMeTAD^+^ and deteriorate the long‐term maintenance of the device performance.^[^ ^27^ ^]^ Strategies of inserting an interlayer to modify the heterojunction between the perovskite and charge transport layers hence is highly desirable to boost optoelectronic properties of PSCs.^[^ ^28^, ^29^, ^30^ ^]^

In this work, we demonstrate a highly efficient hole‐selective heterojunction in the n–i–p planar PSCs, wherein a solution‐processed chromium multioxide (CrO_x_) interlayer is introduced between the perovskite and Spiro‐OMeTAD. Metal oxides are superior in terms of conductivity, wide band gap, and stability than conjugated small molecule and polymers.^[^ ^31^, ^32^, ^33^, ^34^ ^]^ The multivalence state oxides CrO_x_ simultaneously reduce defect sites via redox shuttle at perovskite contact, and decrease the VBM‐HOMO energy offset between the perovskite and Spiro‐OMeTAD to diminish thermionic losses for collecting holes and to promote charge transport at the perovskite/CrO_x_/Spiro‐OMeTAD heterojunction, resulting in the suppression of both defect‐assisted recombination and interface carrier recombination in the PSCs. As a result, we achieve a remarkably improved PCE of 21.21% compared to 18.46% of the control device. It is among the highest efficiencies for polycrystalline MAPbI_3_ based n–i–p planar PSCs reported to date. Moreover, the device stability is significantly enhanced due to the stabilization effects at the heterojunction. The unencapsulated device maintains 90% of its initial efficiency after as long as 500 h storage in 60% relative humidity at room temperature under air, whereas the control device can remain only 10% of its initial efficiency. At such heterojunction, the CrOx enhances the hole transport, avoids the hole accumulation, and impedes the attack of moisture on the perovskite film and the iodine ion migration into the Spiro‐OMeTAD layer. This work highlights a promising strategy of constructing a highly effective charge‐selective heterojunction to further increase efficiency and stability of PSCs.

## 2 Results and Discussion

**Figure 1:**
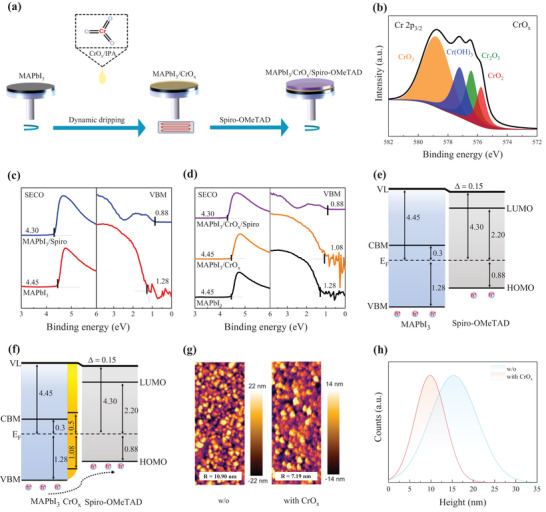
a) Schematics of fabrication processes of a MAPbI_3_/CrO_x_/Spiro‐OMeTAD heterojunction. b) XPS Cr 2p_3/2_ core level spectra of CrO_x_ film. UPS‐derived secondary electron cutoff regions and frontier electronic structure regions of c) MAPbI_3_/Spiro‐OMeTAD and d) MAPbI_3_/CrO_x_/Spiro‐OMeTAD, respectively. e,f) Corresponding energy level alignments. g) Surface morphologies of MAPbI_3_ films with and without CrO_x_. (h) Surface height histograms derived from the AFM images.

**Figure** **1**a presents the fabrication process of a MAPbI_3_/CrO_x_/Spiro‐OMeTAD hole‐selective heterojunction. Detailed film preparations are given in the Experimental Section. X‐ray photoelectron spectroscopy (XPS) is applied to confirm the successful insertion of CrO_3_ thin layer between the perovskite and Spiro‐OMeTAD. The deconvolution of XPS Cr 2p_3/2_ core level spectrum by Gaussian–Lorentzian line shapes demonstrates that the solution‐processed CrO_x_ film in fact composes multivalence state oxide complexes including CrO_3_, Cr(OH)_3_, Cr_2_O_3_, and CrO_2_ (Figure 1b), where the Cr(OH)_3_ constituent originates from hydroxylation in the isopropanol solution.^[^ ^35^ ^]^ The ion pair Cr^6+^ – Cr^3+^ (Cr^4+^) can function as a redox shuttle that oxides Pb^0^ and reduces I^0^ defects via the chemical reactions of Cr^6+^ + Pb^0^ → Pb^2+^ + Cr_3_+ (Cr^4+^), and Cr^3+^ (Cr^4+^) + I^0^ → Cr^6+^ + I^−^ at the perovskite/CrO_x_ interface.^[^ ^36^ ^]^ We then carry out UV photoelectron spectroscopy (UPS) measurement to investigate the interfacial energetics of the MAPbI_3_/CrO_x_/Spiro‐OMeTAD heterojunction (Figure 1c,d). Determined from these UPS spectra, the VBM of MAPbI_3_ and the HOMO of Spiro‐OMeTAD with respect to the Fermi level (*E*_F_) position are 1.28 and 0.88 eV, respectively. It clearly indicates that there is a 0.4 eV VBM‐HOMO energy offset (Figure 1e), which in principle constrains the built‐in field and thus leads to thermionic losses for collecting holes at the MAPbI_3_/Spiro‐OMeTAD interface. After inserting a thin CrO_x_ layer, the VBM of the CrO_x_ is located at 1.08 eV below the E_F_ (Figure 1f), creating a step‐type energy level alignment at the MAPbI_3_/CrO_x_/Spiro‐OMeTAD heterojunction. Such energy level alignment effectively can reduce the thermionic losses for the holes and promote hole transport from MAPbI_3_ to Spiro‐OMeTAD. Considering that the work function of MAPbI_3_/CrO_x_ film (4.45 eV) is same as that of the pristine MAPbI_3_ confirmed by no energy shift of XPS Pb 4f, I 3d and N 1s core level spectra (Figure S1, Supporting Information), we hence conclude that there is no potential step at the perovskite/CrO_x_ interface. This is different from the case of perovskite/thermal‐evaporated molybdenum trioxide interface with the formation of an interface dipole as reported previously.^[^ ^37^ ^]^ Correspondingly, the intrinsic work function of the solution‐processed CrO_x_ film is also 4.45 eV (Figure S2, Supporting Information). Figure 1g shows the surface atomic force microscopy (AFM) measurements of the perovskite films, where the root mean square (RMS) roughness reduces from 10.9 (MAPbI_3_) to 7.2 nm (MAPbI_3_/CrO_x_), and the surface height histogram of the MAPbI_3_/CrO_x_ film exhibits a clear shift toward lower heights, i.e., a smoother surface (Figure 1h). It is believed that a smoother surface is one of the key reasons for improving charge transport at the MAPbI_3_/CrO_x_/Spiro‐OMeTAD heterojunction.^[^ ^38^ ^]^

**Figure 2:**
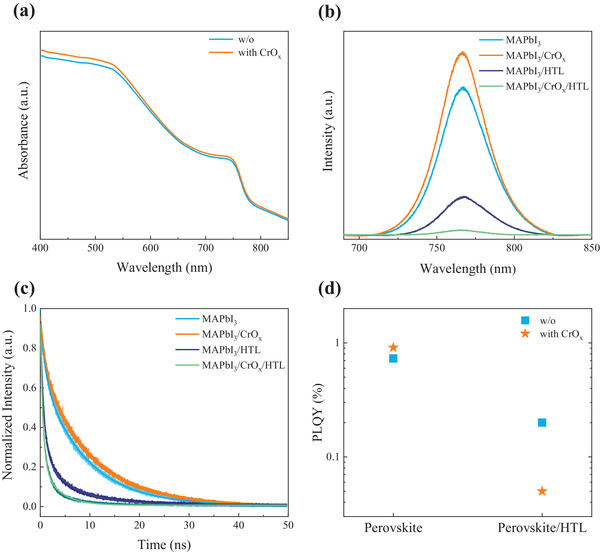
a) UV–vis absorption spectra of the perovskite films with and without CrO_x_. b) Steady‐state PL spectra, and c) Time‐resolved PL decays. d) PLQY results.

**Figure** **2**a displays the UV‐vis absorption spectra of the MAPbI_3_ and MAPbI_3_/CrO_x_ films. The MAPbI_3_/CrO_x_ film has a slight enhancement of the optical absorbance owing to defect reduction at the interface. The unchanged intensities of X‐ray diffraction (XRD) patterns demonstrate that the solution‐processed CrO_x_ does not influence the perovskite film crystallinity (Figure S3, Supporting Information). We further perform steady‐state photoluminescence (PL) and time‐resolved photoluminescence (TRPL) to probe the charge dynamics. When compared to the perovskite films without a charge transport layer, the increased PL intensity (Figure 2b) provides evidence that CrO_x_ helps to suppress nonradiative recombination by eliminating perovskite surface defect sites, which is consistent with a longer lifetime of the perovskite/CrO_x_ film than the control film observed in the TRPL spectra (Figure 2c). For the perovskite films with a hole transport layer of Spiro‐OMeTAD, more quenching is clearly observed (Figure 2b) and its carrier lifetime ( *τ*_1_ = 0.53 *ns*; *τ*_2_ = 3.45 *ns*) is shorter than the control sample ( *τ*_1_ = 0.70 *ns*; *τ*_2_ = 6.01 *ns*) (Figure 2c). It is revealed that CrO_x_ effectively enhances the photogenerated hole transfer from perovskite to Spiro‐OMeTAD, reducing interface charge recombination at the hole‐selective heterojunction. We further use the equation of $\eta_{t}=\left(1-\frac{PLQY_{Per/HTL}}{PLQY_{Per}}\right)$ ^[^ ^39^ ^]^ to quantify the hole transfer efficiency (*η*_*t*_) between the perovskite and Spiro‐OMeTAD. The photoluminescence quantum yields (PLQYs) for the MAPbI_3_ and MAPbI_3_/CrO_x_ are 0.73% and 0.91%, respectively, and the PLQY values for the MAPbI_3_/Spiro‐OMeTAD and MAPbI_3_/CrO_x_/Spiro‐OMeTAD are 0.22% and 0.02%, respectively (Figure 2d). The calculated *η*_*t*_ significantly increases from 69.87% to 97.80% at the heterojunction after inserting CrO_x_.

**Figure 3:**
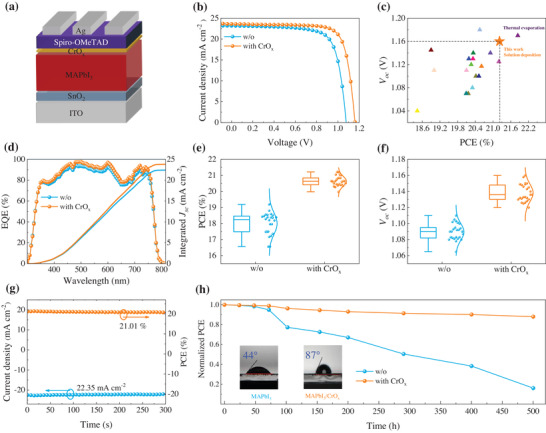
a) Device architecture of n–i–p planar PSCs. b) *J–V* curves of the control and device with CrO_x_ under reverse scan. c) Literature survey of recent achievements on *V*_oc_ and PCE of polycrystalline MAPbI_3_ based n–i–p PSCs. d) EQE spectra and integrated photocurrent density. Statistics of e) PCE and f) *V*_oc_ of the devices. g) Steady‐state photocurrent density and power output at the maximal power point. h) Comparison of normalized efficiency decay of unencapsulated PSCs as a function of storage time in ambient air with 60% relative humidity at room temperature. Insert represents the water angles of perovskite films with and without CrO_x_.

We fabricate n–i–p planar PSCs with a device architecture of ITO/SnO_2_/perovskite/CrO_x_/Spiro‐OMeTAD/Ag using MAPbI_3_ displayed in **Figure** **3**a. The dependence of the device PCE on the CrO_x_ layer thickness is carefully evaluated, and the optimal thickness that produces the champion device is 8 nm (Figures S4,S5; Table S2, Supporting Information). Figure 3b shows the current density‐voltage (*J–V*) curves of the devices under a simulated AM 1.5 G light illumination at 100 Mw cm^–2^. The control device has a typical PCE of 18.46% with an open‐circuit voltage (*V*_oc_) of 1.077 V, a short current density (*J*_sc_) of 23.16 mA cm^–2^ and a fill factor (FF) of 74%. In striking contrast, the champion device with CrO_x_ yields a remarkably improved PCE of 21.21% with a higher *V*_oc_ of 1.161 V, *J*_sc_ of 23.61 mA cm^–2^ and FF of 77.39%. The obtained efficiencies are among the highest PCEs for polycrystalline MAPbI_3_ based n–i–p planar PSCs reported to date (Figure 3c; Table S3, Supporting Information). **Table** **1** summarizes the corresponding photovoltaic parameters of the devices for comparison.

**Table 1:** Photovoltaic parameters of the PSCs with and without CrO_x_ under reverse scan

CrO_x_	*V*_oc_ [V]	*J*_sc_ [mA cm^–2^]	FF [%]	PCE [%]
w/o	1.077	23.16	74.00	18.46
with	1.161	23.61	77.39	21.21

We then characterize external quantum efficiency (EQE) spectra and the integrated photocurrent densities (22.90 mA cm^–2^ for the device with CrO_x_, 22.10 mA cm^–2^ for the control device) closely agree with *J*_sc_ in the *J–V* curves (Figure 3d). The distinct difference in performance between the modified device and the control device is attributed to the improvement of all photovoltaic parameters including *V*_oc_, *J*_sc_, and FF. The statistical charts from the various batches in Figure 3e,f and Figure S6 (Supporting Information) show good reproducibility of the devices. We further investigate the stability of the device with CrO_x_. The modified device has a stabilized photocurrent density of 22.35 mA cm^–2^ and a stabilized power output of 21.01% at continuous operation (AM 1.5 G illumination) at the maximum power point for 300 s (Figure 3g). Under ambient air in 60% relative humidity at room temperature, the unencapsulated device with CrO_x_ retains 90% of its initial efficiency after up to 500 h storage, whereas the control device has only 10% of its initial PCE left (Figure 3h). The enhanced stability can be ascribed to the reduced perovskite surface defects and the increased hydrophobicity of the perovskite film coated with CrO_x_ as the water contact angle largely increases from 44° to 87°. These results indicate that the CrO_x_ not only boosts the hole transport across the heterojunction, but also functions as a self‐encapsulation layer to impede the attack of moisture on the underlying perovskite film and the penetrability of iodine ion into Spiro‐OMeTAD.

**Figure 4:**
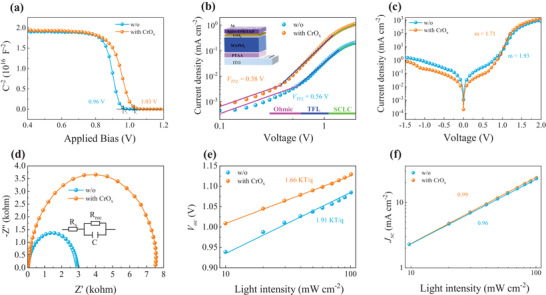
a) Mott–Schottky plots of PSCs. b) Dark *J–V* curves of hole‐only devices. c) Dark *J–V* characteristics of PSCs. The ideality factor (m) is extracted from the diffusion‐dominated current region according to the equation of $m=(\frac{kT}{q}\frac{d\ln J}{dV}$)^–1^.^[^ ^46^ ^]^ d) EIS Nyquist plots. e) Dependence of *V*_oc_ on light intensity and f) Dependence of *J*_sc_ on light intensity of PSCs.

To obtain more insight on the improved performance of our PSCs, we carry out the capacitance–voltage (*C–V*) curves to extract the built‐in potential (*V*_bi_) of the devices according to the Mott–Schottky equation:^[^ ^40^ ^]^ $\frac{1}{C^{2}}=\frac{2(V_{bi}-V)}{A^{2}e\varepsilon \varepsilon_{0}N_{A}}$, where *A* is device area, *N*_A_ is charge carrier density, *ε*_0_ is vacuum permittivity, and *ε* is relative permittivity. A higher *V_bi_* of 1.03 V for the modified device is observed than 0.96 V for the control device, which is in accordance with the observed *V*_oc_ enhancement (**Figure** **4**a). We ascribe the increased flat band potential to the reduced VBM‐HOMO energy offset between the perovskite and Spiro‐OMeTAD that diminishes thermionic losses for collecting holes at the heterojunction and thus reduces the device energy loss. We then estimate charge trap density and mobility using the space‐charge‐limited current (SCLC) method.^[^ ^41^ ^]^ As determined from the dark *J–V* curves of hole‐only device with a structure of ITO/PTAA/MAPbI_3_/CrO_x_/Spiro‐OMeTAD/Ag (Figure 4b), the calculated hole trap density decreases from 9.04 × 10 ^15^ cm^–3^ for the control device to 4.52 × 10 ^15^ cm^–3^ for the modified device, and the corresponding hole mobility increases from 1.12 × 10^–3^ to 5.60 × 10^–3^ cm^2^ V^–1^ s^–1^. These results demonstrate that the defect density is greatly reduced and the charge transport is significantly improved across the perovskite/CrO_x_/Spiro‐OMeTAD heterojunction. We also characterize the dark *J–V* curves of the full PSC device (Figure 4c). Both the leakage current at negative bias and the ideality factor (m) at positive bias of the modified device are smaller than those of the control device, suggesting suppressed nonradiative recombination. This is in agreement with the increased recombination resistance (*R*_rec_) fitted in the electrical impedance spectroscopy (EIS) in Figure 4d and Table S4 (Supporting Information).

To further elucidate the charge recombination mechanisms of the improved devices, we provide the light intensity (*P*) – dependent *V*_oc_ and *J*_sc_, as displayed in Figure 4e,f, respectively. The slope of the dependence of *V*_oc_ versus *P* is applied to evaluate the degree of trap‐assisted recombination via the equation:^[^ ^42^ ^]^ $V_{oc}=\frac{nKT}{q\ln(P)}$, where *K* is Boltzmann constant and *T* is absolute temperature. Compared with 1.91 KT q^−1^ for the control device, the slope of the device with CrO_x_ decreases to 1.66 KT q^−1^, revealing that the trap‐assisted recombination is effectively suppressed due to the reduced defect density. The relationship between *J*_sc_ and P can be expressed by *J_sc_* ∝ *P^*α*^*, where the exponential factor *α* represents the extent of biomolecular recombination.^[^ ^43^ ^]^ The extracted *α* of the modified device increases to 0.99 compared to 0.96 of the control device, indicating approximatively a minimal interface charge recombination at the contact between perovskite and Spiro‐OMeTAD after inserting CrO_x_ owing to the diminished VBM‐HOMO offset and the improved hole extraction capability from perovskite to Spiro‐OMeTAD. Notably, this is different from the formation of an interface dipole at the perovskite/thermal‐evaporated molybdenum trioxide interface that reinforces the built‐in field and prevents the photogenerated charges from recombining.^[^ ^37^ ^]^ The above results suggest that the perovskite/CrO_x_/Spiro‐OMeTAD heterojunction effectively suppresses the nonradiative recombination in the PSCs, leading to a better hole transport. Additionally, it is expected that the CrO_x_ also acts as a charge generation layer due to spontaneous charge transfer from intrinsic defect states in oxides via thermal diffusion and the internal electric‐field at the CrO_x_/Spiro‐OMeTAD interface.^[^ ^44^, ^45^ ^]^ The generated holes from CrOx inject into the neighboring Spiro‐OMeTAD, which synchronously promotes the performance of the device.

## 3 Conclusion

In summary, we have demonstrated the roles of perovskite/CrO_x_/Spiro‐MeTAD hole‐selective heterojunction in developing high performance n–i–p planar PSCs. The solution‐processed CrO_x_ composes multivalence state oxide complexes. The perovskite/CrO_x_/Spiro‐MeTAD heterojunction not only effectively reduces the defect sites via a redox shuttle at the perovskite contact, but also decreases VBM‐HOMO offset between perovskite and Spiro‐OMeTAD. These result in diminishing thermionic losses for collecting holes at the heterojunction, which thus promotes charge transport, and simultaneously suppresses both defect‐assisted recombination and interface carrier recombination. As a result, we successfully achieve a PSC with a significantly improved PCE of 21.21% compared to 18.46% of the control device. Our efficiency is among the highest PCEs for polycrystalline MAPbI_3_ based n–i–p planar PSCs reported to date. These findings of this work provide new insight into the improved performance of the PSCs via constructing highly efficient charge‐selective heterojunctions.

## 4 Experimental Section

### Materials

Lead iodide (PbI_2_, 99.99%), 2,2″,7,7″‐Tetrakis[*N*,*N*‐di(4‐methoxyphenyl)amino]‐9,9'‐spirobifluorene (Spiro‐OMeTAD, 99.8%), bis(trifluoromethanesulfonyl)imide lithium (Li‐TFSI, 99.95%), and tertbutylpyridine (tBP, 96%) were received from Polymer Light Technology Corp. Methylammonium iodide (MAI, 99.5%) was provided by Greatcell Solar Ltd. Stannic oxide (SnO_2_) colloid precursor (15% in H_2_O colloidal dispersion) and chromium trioxide powder (99.99%) were purchased from Alfa Aesar. *N*, *N*‐Dimethylformamide (DMF, 99.8%), dimethyl sulfoxide (DMSO, 99.8%), isopropyl alcohol (IPA, 99.5%), acetonitrile, and chlorobenzene (CB, 99.5%) were obtained from Sigma–Aldrich. All materials were used as received.

### Device Fabrication and Characterizations

Indium tin oxide (ITO)‐coated glasses were washed by sequentially sonicating in deionized water, acetone, and ethanol for 30 min each step. The dried ITO was treated by UV–ozone for 20 min before use. The SnO_2_ colloid precursor was diluted using ultrapure water (1:6 in volume ratio) and then spin‐coated on the ITO at 3000 rpm for 30 s, and was annealed on a hotplate at 150 °C for 30 min in air. The MAPbI_3_ perovskite precursor was prepared by dissolving 497.8 mg PbI_2_ and 159.0 mg MAI in the mixed solvent of DMF/DMSO (750 µL:85 µL). The perovskite film was spin‐coated on the electron transport layer SnO_2_ at 4000 rpm for 30 s in a N_2_‐filled glove‐box. 120 µL CB was dripped at the center of the film after 7 s spin coating, and the perovskite film was annealed at 105 °C for 10 mins. The filtered CrO_x_ solution in IPA with the different concentrations was spin‐coated on the perovskite film at 6000 rpm for 30 s, following by 100 °C annealing for 10 min. The hole transport layer spiro‐OMeTAD (73 mg spiro‐OMeTAD in 28.8 µl tBP and 17.5 µl Li‐TFSI solution with 520 mg ML^–1^ in acetonitrile) was deposited on the perovskite film via spin‐coating at 4000 rpm for 30 s. Finally, a 100 nm silver layer was deposited by thermal evaporation with a mask at a pressure of 10^–6^ mbar. The *J–V* curves of the devices were measured in the N_2_‐filled glovebox using a Keithley 2400 system under a solar simulator (SS‐F5‐3A, Enlitech). The light intensity (100 mW cm^–2^) was calibrated by a certified standard silicon solar cell with KG‐5 filter (SRC‐2020, Enlitech). The EQE spectra were recorded by QE‐R system (Enlitech). The capacitance‐voltage curve was conducted by an impedance spectroscope (PGSTAT302N, Autolab). The EIS measurement was performed via a precision impedance analyzer (Agilent 4294A) under a bias of 1 V.

### Film Characterizations

The surface roughness of the film was measured by AFM (Asylum Research, Oxford). The XRD patterns were recorded using PANalytical X‐ray diffractometer with Cu K*α* radiation. The UV–vis absorption spectra were collected by UV/vis/NIR spectrometer (TU‐1901). The steady‐state PL spectra were recorded with a fluorescence spectrometer (PerkinElmer LS 55) using a 470 nm excitation light source. The time‐resolved PL decay was measured via a time‐corrected single photon counting system at an excitation wavelength of 373 nm. The PLQY was collected on a fluorometer equipped with an integrating sphere at a wavelength of 470 nm (FS‐5, Edinburgh).

### Photoelectron Spectroscopy Measurements

UPS/XPS measurements were performed in an ultrahigh vacuum surface analysis system equipped with a fast entry load‐lock, a transfer chamber, a preparation chamber and an analysis chamber with a base pressure of 10^–10^ mbar. A Scienta R3000 spectrometer was equipped to detect the photoelectron kinetics. UPS was employed with the HeI 21.22 eV as the excitation source and an energy resolution of 50 meV. The work function and VBM were derived from the secondary electron cutoff and the frontier edge of the occupied density states, respectively. The precise VBM position of the perovskite film was determined in a logarithmic intensity scale due to its strong band dispersion, and the VBM position of Spiro‐OMeTAD was determined in a linear intensity scale. XPS was carried out with a monochromatic Al K*α* 1486.6 eV as the excitation source. All recorded spectra were calibrated by referring to the Fermi level edge and Au 4f_7/2_ position of the Ar^+^ ion sputter‐cleaned Au film.

## Conflict of Interest

The authors declare no conflict of interest.

## Supporting information

Supporting Information

## Data Availability

The data that support the findings of this study are available from the corresponding author upon reasonable request.
