# Supplementary material for: Constructing Chromium Multioxide Hole‐Selective Heterojunction for High‐Performance Perovskite Solar Cells
Source: Adv Sci (Weinh). 2022 Aug 28;9(30):2203681. doi: 10.1002/advs.202203681 (PMC9596853; doi:10.1002/advs.202203681)
Supplement: Supplementary file 1 — Supporting Information [file ADVS-9-2203681-s001.pdf]

ADVANCED SCIENCE
Open Access

Supporting Information

for *Adv. Sci.*, DOI 10.1002/advs.202203681

Constructing Chromium Multioxide Hole-Selective Heterojunction for High-Performance Perovskite Solar Cells

Sheng Jiang, Shaobing Xiong, Wei Dong, Danqin Li, Yuting Yan, Menghui Jia, Yannan Dai, Qingbiao Zhao, Kai Jiang, Xianjie Liu, Liming Ding*, Mats Fahlman, Zhenrong Sun and Qinye Bao**

Supporting Information

Constructing Chromium Multioxide Hole-Selective Heterojunction for High-performance Perovskite Solar Cells

Sheng Jiang, Shaobing Xiong, Wei Dong, Danqin Li, Yuting Yan, Menghui Jia, Yannan Dai, Qingbiao Zhao, Kai Jiang, Xianjie Liu, Liming Ding*, Mats Fahlman, Zhenrong Sun, Qinye Bao**

S. Jiang, S. Xiong, D. Li, Y. Yan, Y. Dai, K. Jiang, Prof. Q. Zhao, Prof. Q. Bao
School of Physics and Electronic Science, East China Normal University, Shanghai 200241, China
E-mail: kjiang@ee.ecnu.edu.cn
qybao@clpm.ecnu.edu.cn

W. Dong
Shanghai Key Laboratory of Magnetic Resonance, East China Normal University, Shanghai 200241, China

M. Jia, Prof. Z. Sun
State Key Laboratory of Precision Spectroscopy, East China Normal University, Shanghai 200241, China

Prof. X. Liu, Prof. M. Fahlman
Laboratory of Organic Electronics, ITN, Linköping University, Norrköping SE-60174, Sweden

Prof. L. Ding
Center for Excellence in Nanoscience (CAS), Key Laboratory of Nanosystem and Hierarchical Fabrication (CAS), National Center for Nanoscience and Technology, Beijing 100190, China
Email: ding@nanoctr.cn

Prof. Q. Bao
Collaborative Innovation Center of Extreme Optics, Shanxi University, Taiyuan, Shanxi 030006, China

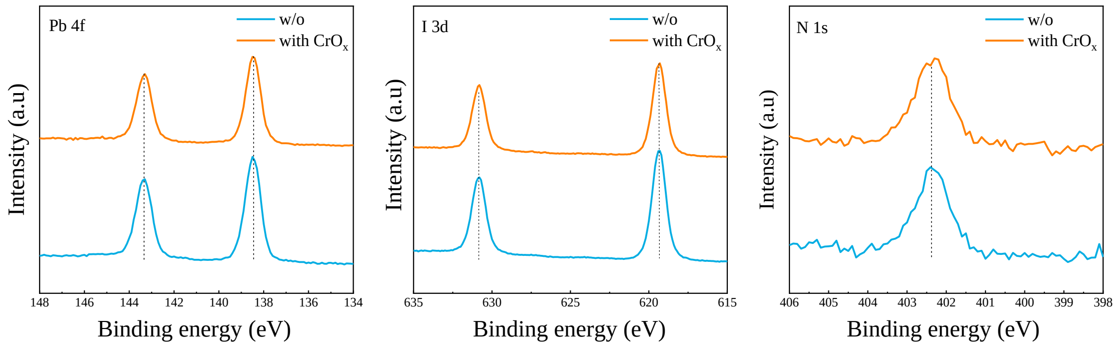

Figure S1. XPS Pb 4f, I 3d and N 1s core level spectra of the $MAPbI_3$ films with and without CrO_x.

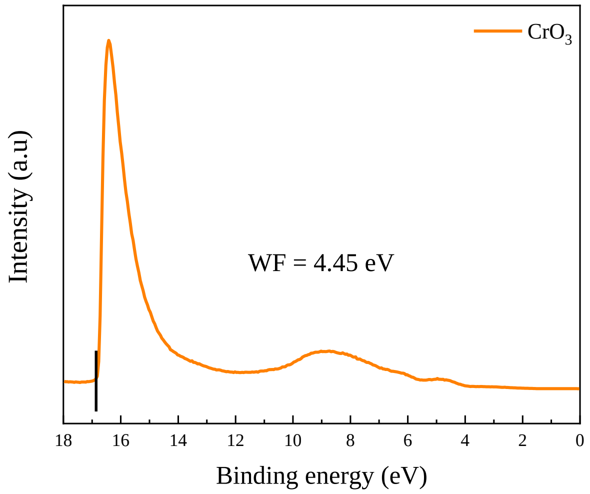

Figure S2. UPS spectrum of the CrO_x film.

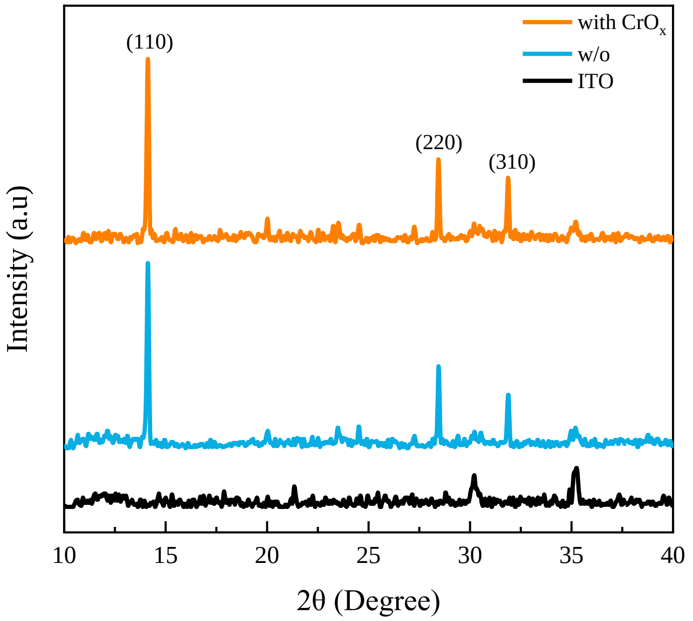

Figure S3. XRD patterns of ITO substrate, and the $MAPbI_3$ films with and without CrO_x.

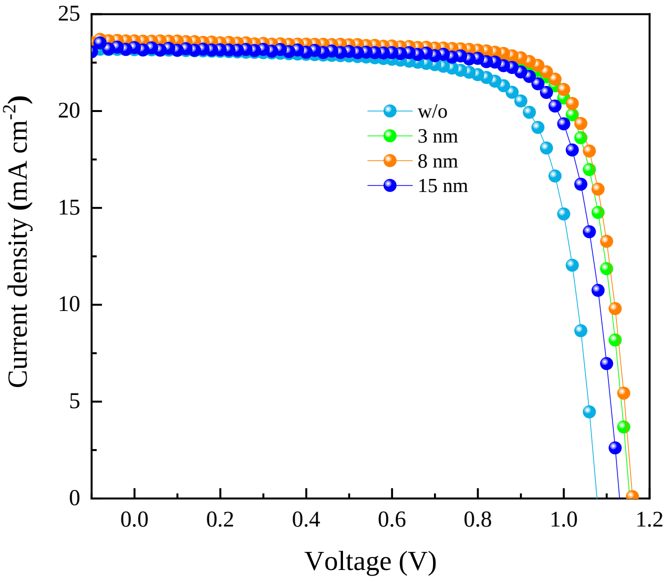

Figure S4. *J-V* curves of the PSCs with different thicknesses of CrO_x.

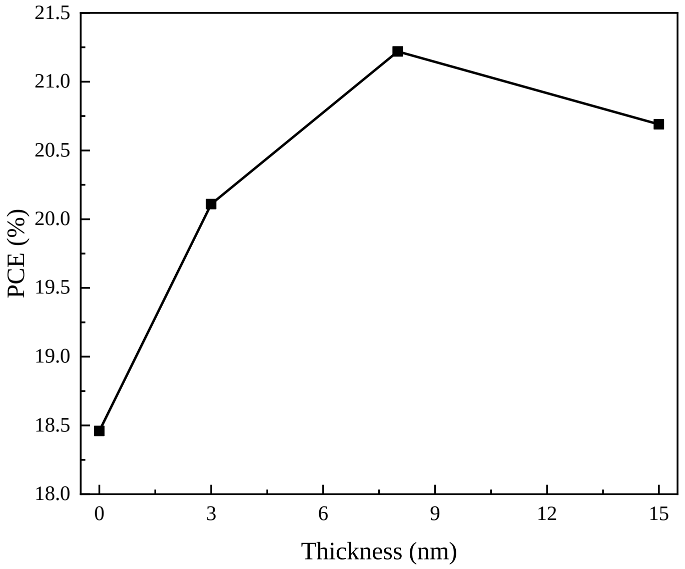

Figure S5. PCE evolution of the PSCs with different thicknesses of CrO_x.

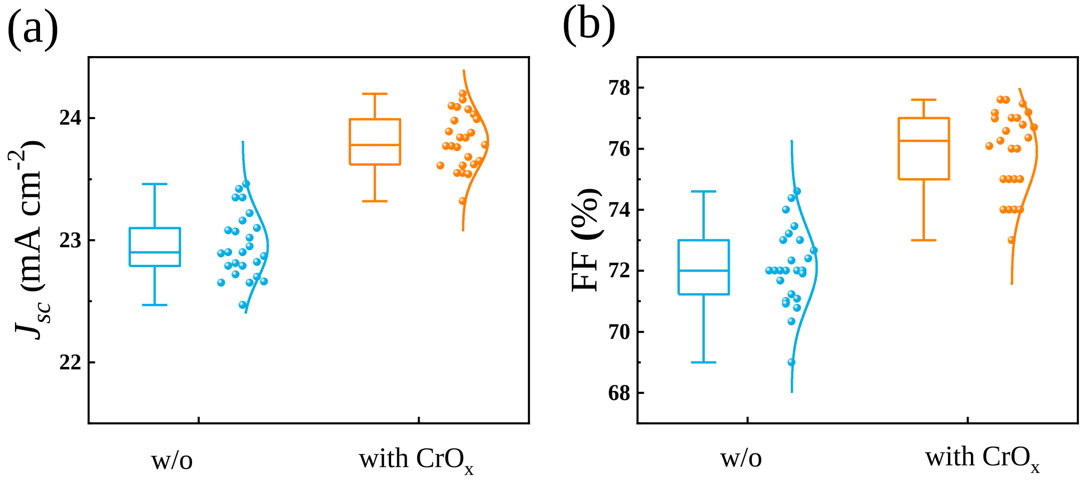

Figure S6. Statistics of (a) J_{sc} and (b) FF of the PSCs.

Table S1. Parameters of the fitted TRPL spectra.

Sample	A_1 (%)	τ_1 (ns)	A_2 (%)	τ_2 (ns)	τ_{ave} (ns)
$MAPbI_3$	31	1.07	69	8.21	7.81
$MAPbI_3/CrO_x$	27	1.06	73	9.92	9.58
$MAPbI_3$/HTL	70	0.70	30	6.01	4.87
$MAPbI_3/CrO_x$/HTL	74	0.53	26	3.45	2.56

The TRPL curves are fitted by the bi-exponential decay model：

$y = A_1 \exp\left(-\frac{x}{\tau_1}\right) + A_2 \exp\left(-\frac{x}{\tau_2}\right) + y_0$, where τ_1 and τ_2 are the lifetimes for the fast and slow recombination, respectively.

The average lifetime τ_{avg} is calculated by the following equation:

$\tau_{avg}=(A_1\tau_1{}^2 + A_2\tau_2{}^2)/(A_1\tau_1 + A_2\tau_2)$

Table S2. Photovoltaic parameters of the PSCs with different thicknesses of CrO_x.

CrO_x (nm)	V_{oc} (V)	J_{sc} (mA cm^{-2})	FF (%)	PCE (%)
0	1.077	23.16	74.00	18.46
3	1.153	23.51	77.03	20.88
8	1.161	23.61	77.39	21.21
15	1.130	23.27	76.48	20.11

Table S3. Recent achievements for polycrystalline $MAPbI_3$ based n-i-p PSCs.

Device structure	V_{oc} (V)	J_{sc} (mA cm^{-2})	FF (%)	PCE (%)	Ref.
FTO/SnO_2/$MAPbI_3$/Spiro-OMeTAD/Au	1.10	22.98	81.27	20.52	[1]
ITO/SnO_2/$MAPbI_3$/Spiro-OMeTAD/Au	1.13	24.07	76.80	20.93	[2]
ITO/SnO_2/$MAPbI_3$-$CsPbBr_3$ /Spiro-OMeTAD/Au	1.11	23.57	76.88	20.17	[3]
FTO/Chol-SnO_2/$MAPbI_3$/Spiro-OMeTAD/Au	1.14	22.80	72.41	18.90	[4]
FTO/ZnO/ZnS/$MAPbI_3$/Spiro-OMeTAD/Au	1.12	23.39	77.70	20.26	[5]
FTO/Li–SnO_2/$MAPbI_3$/ Spiro-OMeTAD/Au	1.13	23.24	76.66	20.18	[6]
FTO/SnO_2/TiO_2/$MAPbI_3$/WSe_2/Spiro-OMeTAD/Ag	1.12	23.33	80.70	21.18	[7]
ITO/SnO_2/KF/$MAPbI_3$/Spiro-OMeTAD/Ag	1.14	23.17	77.00	20.33	[8]
ITO/$Ti_3C_2T_x$/SnO_2/$MAPbI_3$/Spiro-OMeTAD/Ag	1.07	23.88	78.40	20.09	[9]
ITO/SnO_2/ABSA/$MAPbI_3$/Spiro-OMeTAD/MoO_3/Ag	1.13	22.93	78.75	20.32	[10]
ITO/SnO_2/TA/$MAPbI_3$/Spiro-OMeTAD/MoO_x/Ag	1.11	23.03	80.12	20.61	[11]
FTO/CL-TiO_2/$MAPbI_3$/Spiro-OMeAD/Ag	1.04	23.90	74.00	18.43	[12]
FTO/SnO_2/$MAPbI_3$/Spiro-OMeTAD/Au	1.14	22.20	75.75	19.24	[13]
FTO/SnO_2/$MAPbI_3$/MoO_x (thermal evaporation)/Spiro-OMeTAD/Ag	1.18	22.97	80.11	21.70	[14]
FTO/SnO_2/$MAPbI_3$/Pc/Spiro-OMeTAD/Au	1.03	22.75	76.55	18.01	[15]
FTO/SnO_2/$MAPbI_3$/Spiro-OMeTAD/Au	1.18	22.42	77.00	20.55	[16]
FTO/SnO_2/$MAPbI_3$/Spiro-OMeTAD/Au	1.13	22.57	77.77	20.14	[17]
ITO/SnO_2/$MAPbI_3$/Spiro-OMeTAD/Ag	1.14	22.72	80.00	20.91	[18]
ITO/SnO_2/$MAPbI_3$/CrO_x/Spiro-OMeTAD/Ag	**1.16**	**23.61**	**77.39**	**21.22**	**This work**

Table S4. Fitted parameters of EIS plots

CrO_x	R_s (ohm)	R_{rec} (Kohm)
w/o	31.9	2.8
with	28.1	7.8

References

[1] C. Chen, Y. Jiang, J. Guo, X. Wu, W. Zhang, S. Wu, X. Gao, X. Hu, Q. Wang, G. Zhou, Y. Chen, J. Liu, K. Kempa, J. Gao, Adv. Funct. Mater. 2019, 29, 1900557.

[2] S. Kim, I. Jeong, C. Park, G. Kang, I. K. Han, W. Kim, M. Park, Sol. Energy Mater. Sol. Cells 2019, 203, 110197.

[3] Y. Yao, P. Hang, P. Wang, L. Xu, C. Cui, J. Xie, K. Xiao, G. Li, P. Lin, S. Liu, D. Xie, S. Che, D. Yang, X. Yu, Nanotechnology 2019, 31, 085401.

[4] J. Yan, Z. Lin, Q. Cai, X. Wen, C. Mu, ACS Appl. Energy Mater. 2020, 3, 3504.

[5] X. Huang, R. Chen, G. Deng, F. Han, P. Ruan, F. Cheng, J. Yin, B. Wu, N. Zheng, J. Am. Chem. Soc. 2020, 142, 6149.

[6] K. Jung, W. S. Chae, J. W. Choi, K. C. Kim, M. J. Lee, J. Energy Chem 2021, 59, 755.

[7] Z. Qin, Y. Chen, X. Wang, X. Liu, Y. Miao, Y. Zhao, J. Phys. Chem. Lett. 2021, 12, 6883.

[8] P. Xu, H. He, J. Ding, P. Wang, H. Piao, J. Bao, W. Zhang, X. Wu, L. Xu, P. Lin, X. Yu, C. Cui, ACS Appl. Energy Mater. 2021, 4, 10921.

[9] L. Yang, B. Wang, C. Dall'Agnese, Y. Dall'Agnese, G. Chen, A. K. Jena, X. Wang, T. Miyasaka, ACS Sustainable Chem. Eng. 2021, 9, 13672.

[10] Y. Sun, J. Zhang, H. Yu, J. Wang, C. Huang, J. Huang, Chem. Eng. J. 2021, 420, 129579.

[11] J. Zhang, H. Yu, J. Mater. Chem. A 2021, 9, 4138.

[12] L. Wang, M. Shahiduzzaman, E. Y. Muslih, M. Nakano, M. Karakawa, K. Takahashi, K. Tomita, J. M. Nunzi, T. Taima, Nano Energy 2021, 86, 106135.

[13] Y. Zou, S. Yuan, A. Buyruk, J. Eichhorn, S. Yin, M. A. Reus, T. Xiao, S. Pratap, S. Liang, C. L. Weindl, W. Chen, C. Mu, I. D. Sharp, T. Ameri, M. Schwartzkopf, S. V. Roth, P. Muller Buschbaum, ACS Appl. Mater. Interfaces 2022, 14, 2958.

[14] F. Wang, Y. Zhang, M. Yang, D. Han, L. Yang, L. Fan, Y. Sui, Y. Sun, X. Liu, X. Meng, J. Yang, Adv. Funct. Mater. 2020, 31, 2008052.

[15] G. Qu, D. Khan, F. Yan, A. Atsay, H. Xiao, Q. Chen, H. Xu, I. Nar, Z. Xu, J.

Energy Chem 2022, 67, 263.

[16] Y. Wang, Y. Liang, Y. Zhang, W. Yang, L. Sun, D. Xu, Adv. Funct. Mater. 2018, 28, 1801237.

[17] Y. Chen, X. Zuo, Y. He, F. Qian, S. Zuo, Y. Zhang, L. Liang, Z. Chen, K. Zhao, Z. Liu, J. Gou, S. F. Liu, Adv. Sci 2021, 8, 2001466.

[18] S. Jiang, C. C. Wu, F. Li, Y. Q. Zhang, Z.H. Zhang, Q. H. Zhang, Z. J. Chen, B. Qu, L. X. Xiao, M. L. Jiang, Rare Met. 2020, 40, 1698.
